# Role of preoperative transarterial chemoembolization (TACE) in intermediate‐stage hepatocellular carcinoma (Hong Kong liver cancer stage IIB)

**DOI:** 10.1002/wjs.12420

**Published:** 2024-12-11

**Authors:** Kunal Nandy, Gurudutt P. Varty, Shraddha Patkar, Tanvi Shah, Kaival Gundavda, Kunal Gala, Nitin Shetty, Suyash Kulkarni, Mahesh Goel

**Affiliations:** ^1^ Division of Gastrointestinal and Hepatobiliary Surgery, Department of Surgical Oncology Tata Memorial Hospital Homi Bhabha National Institute Mumbai India; ^2^ Department of Interventional Radiology Tata Memorial Hospital Homi Bhabha National Institute Mumbai India

**Keywords:** hepatectomy, hepatocellular carcinoma, Hong Kong Liver Cancer staging, preoperative TACE, transarterial chemoembolisation

## Abstract

**Introduction:**

Transarterial chemoembolization (TACE) has an established role in advanced HCC. The present study evaluates the role of TACE as a neoadjuvant modality in the management of intermediate HCC [Hong Kong Liver Cancer (HKLC) stage IIB].

**Materials and methods:**

A retrospective analysis of HCC patients treated between January 2010 and August 2022 was performed. Patients belonging to intermediate‐stage HCC (HKLC IIB) were divided into two groups, upfront surgery (UPS) and post‐TACE (pTACE). Propensity score matching was done, and the primary endpoint of the study was overall survival (OS).

**Results:**

A total of 247 patients of HKLC IIB were identified during this period. Of these, 77 patients in each group were considered for analysis after propensity matching. The median follow‐up was 36.4 months (0.46–144.26). In the propensity matched population (*n* = 154), on an intention‐to‐treat analysis, the median OS of the UPS group and the pTACE group was 30.06 and 39.26 months, respectively (*p* value = 0.77). In patients who underwent curative resection, the median OS of the UPS group was 30.68 versus 90.97 months in the pTACE group (*p* value = 0.006) and median DFS was 13.56 months for the UPS group versus 44.02 months in the pTACE group, respectively (*p* value = 0.013).

**Conclusion:**

In intermediate‐stage hepatocellular carcinoma (HKLC IIB), pTACE can be used to better select patients with borderline resectability. Survival was significantly improved in patients who received pTACE and were able to undergo surgical resection.

## Highlights

- HKLC stage IIB patients as per guidelines should undergo upfront surgery but not all of them are fit to undergo the same.
- Preoperative transarterial chemoembolisation (pTACE) was done in the HKLC stage IIB for downsizing, to rule out multicentricity, tumor bleeding, and in unfit patients.
- On intention‐to‐treat analysis, survival was similar in patients who received pTACE or upfront surgery, and patients who underwent curative surgery after pTACE had better survival than the propensity matched group who underwent upfront surgery.

## 1 INTRODUCTION

The principle of transarterial chemoembolization (TACE) in the treatment of HCC is attributed to the predominant hepatic arterial blood supply of the tumor.^1^ TACE has an established role in advanced HCC as recommended in the Barcelona clinic liver cancer stage (BCLC) and Hong Kong liver cancer (HKLC) stages IIIA and IIIB. Surgery is the modality of choice for HKLC IIB.^2^, ^3^ However, there is a subgroup of patients in stage IIB who may not be suitable for upfront surgical resection due to inadequate/borderline future liver remnant (FLR), multicentric disease, or poor performance status. Some of the potential advantages of using TACE in a preoperative setting include tumor downsizing, detection of multicentricity, prevention of intraoperative tumor dissemination, and assessment of tumor biology.^4^, ^5^, ^6^, ^7^ Our previous publication demonstrated the feasibility and utility of TACE in a preoperative setting in a select group of patients.^8^ The present study assesses the effects of preoperative TACE on survival in patients with intermediate‐stage HCC (HKLC stage IIB disease).

## 2 MATERIALS AND METHODS

A retrospective analysis of a prospectively maintained database of all patients with HCC who presented to our center between January 2010 and August 2022 was performed. Patients managed with a curative intent with either upfront surgical resection (UPS) or after preoperative TACE (pTACE) were included in the study. The study protocol conformed to the ethical guidelines of the “World medical association declaration of helsinki—Ethical principles for medical research involving human subjects”.^9^

Decisions regarding treatment plans were taken in a dedicated multidisciplinary ‘Liver clinic’ comprising Hepato‐pancreato‐biliary (HPB) surgical oncologists, interventional radiologists, hepatologists, medical oncologists, and radiation oncologists. All patients underwent preoperative evaluation including blood investigations, tumor markers [carcinoembryonic antigen (CEA), cancer antigen (CA 19‐9), and alpha fetoprotein (AFP) levels], serology, calculation of modified Child–Turcotte–Pugh (CTP) score, a triple‐phase contrast‐enhanced computed tomography (CECT) or gadolinium‐enhanced magnetic resonance imaging (MRI) of the liver, and a CT thorax for staging. Patients were staged according to BCLC and HKLC staging systems.^2^, ^3^, ^10^

The diagnosis of HCC was made on characteristic radiological features of arterial enhancement and venous phase washout. Equivocal radiological findings warranted a biopsy for confirmation. Assessment of cirrhosis was done on CT/MRI with liver contour irregularities, caudate hypertrophy, and the presence of collaterals. Cirrhotic patients underwent upper gastrointestinal endoscopy to look for stigmata of portal hypertension.

Tumor burden score (TBS) was calculated by applying the Pythagorean formula [TBS^2^ = (maximum tumor diameter)^2^ + (number of tumors)^2^] on preoperative imaging data.^11^

**FIGURE 1:**
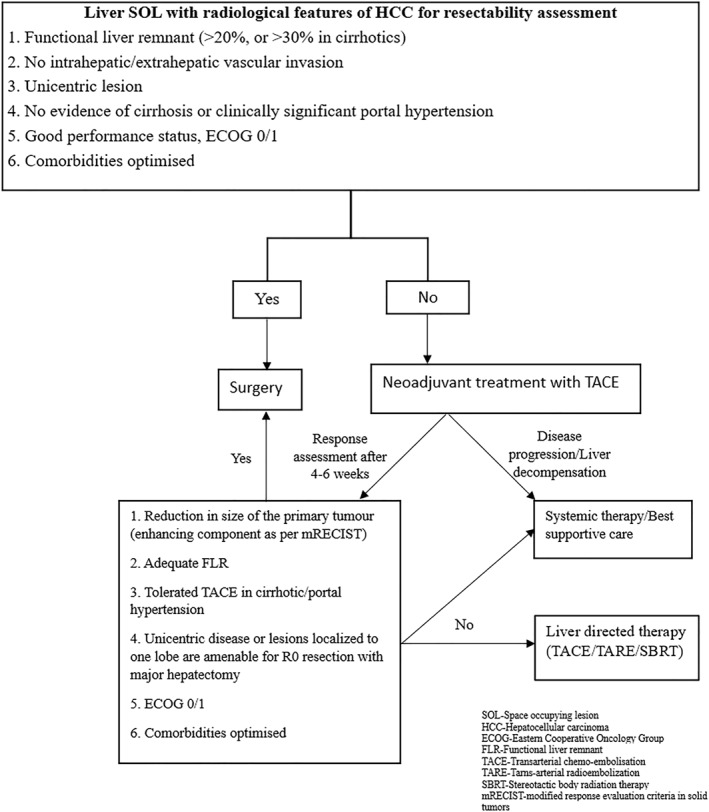
Algorithm for criteria used for preoperative TACE in a patient presenting with liver space‐occupying lesion with radiological features of HCC for resectability assessment.

TACE was considered in the following patients with intermediate‐stage HCC as per the institutional protocol [Figure 1].

1. Downsizing the tumor and achieving adequate FLR. If pTACE was insufficient in achieving the desired minimum FLR, then a portal vein embolization (PVE) was done.
2. To rule out multicentricity especially in cirrhotic patients if there was doubt of a tumor nodule/dysplastic nodule (<1 cm) in the contralateral lobe.
3. Patients with comorbidities who need optimization before resection
4. Tumor bleed/rupture
5. Presence of vascular invasion (infiltration or thrombosis) of intrahepatic portal vein and hepatic veins
6. Patients with clinically significant portal hypertension (CSPH) with borderline FLR (<30%)

The following patients were excluded:

1. HKLC stages I, IIA, III, and IV
2. Metastatic disease at presentation
3. Recurrent disease
4. Cirrhotic patient with a CTP score ≥8
5. Main portal vein thrombosis or invasion

TACE was performed using a standard femoral approach. Drug‐eluting beads (Bio‐compatibles UK, Surrey, UK) 300–500 μm in size, with a dose of 50–75 mg of doxorubicin, were injected. In some patients, conventional TACE was also done with 50 mg of doxorubicin and 10 mL of lipiodol. Response to therapy was evaluated by contrast‐enhanced CT/MRI using the modified Response Evaluation Criteria in Solid Tumors (mRECIST).^12^

After ruling out distant metastasis on abdominal exploration, an intraoperative ultrasound (IOUS) of the liver was performed in all patients to identify previously undetected lesions and to assess the relation of the tumor to major vascular structures. Hypotensive anesthesia and portal triad clamping (Pringle maneuver) were selectively utilized. Parenchymal transection was performed predominantly using a cavitron ultrasonic surgical aspirator (CUSA) along with either water jet, ligasure, or harmonic scalpel as per the surgeon's discretion. Postoperative complications were recorded based on International Study Group of Liver Surgery (ISGLS) criteria and as well as the Clavien–Dindo classification.^13^, ^14^, ^15^, ^16^ All the patients were followed up at three monthly intervals regularly for first 2 years and 6 monthly after that.

Statistical analyses was performed in an intention‐to‐treat manner to compare UPS to pTACE as a primary treatment in intermediate stage HCC. To minimize bias between the pTACE group and the UPS group, a propensity score matching was used. The clinical variables obtained at the time of initial diagnosis and considered to have influenced the decision concerning the primary treatment were used for the 1:1 matching with match tolerance kept at 0.05. The categorical variables were analyzed using Pearson's χ2 test, whereas continuous variables were analyzed using the Mann–Whitney *U* test.The primary endpoint of the study was overall survival (OS). OS was defined as the time interval between the start of treatment (i.e., neoadjuvant therapy or surgery) and the last follow‐up or death. Disease‐free survival (DFS) was defined as the time interval between the start of treatment and the first appearance of recurrence after surgery. Survival curves were plotted using the Kaplan–Meier method and were analyzed using the log‐rank test. Multivariate Cox regression analysis was performed to evaluate factors affecting OS. A *p*‐value of less than 0.05 was considered statistically significant. Statistical analyses were performed using the Statistical Product and Service Solutions (SPSS), version 25.0, for Windows (SPSS Inc., Chicago, IL, USA).

## 3 RESULTS

**FIGURE 2:**
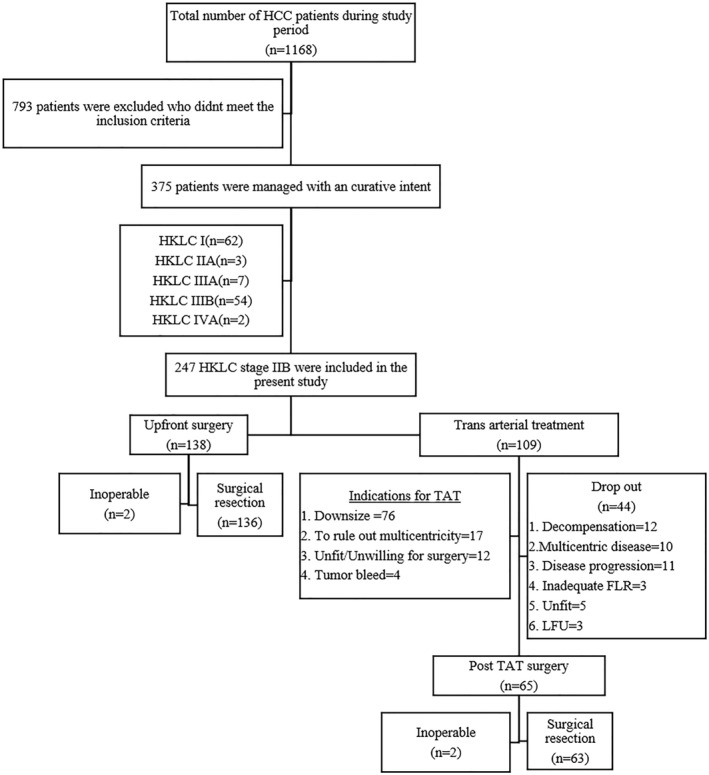
Consort diagram for patient selection.

A total of 1168 patients were evaluated for the study as shown in Figure 2. After eliminating the patients who did not meet the inclusion criteria, 375 patients of HCC were managed with curative intent. Of these 375 patients, 247 patients (baseline cohort) of intermediate‐stage HCC (HKLC stage IIB) were included in the study. The demographic details and tumor characteristics of the patients are shown in Table 1. In the overall population, the distribution of cirrhosis, portal hypertension, and viral markers was significantly different [Table 1]. After 1:1 propensity matching, out of 154 patients, 77 each in upfront surgery (UPS) and post‐TACE (pTACE) groups were selected for analysis [Table 1].

**TABLE 1:** Characteristics of both groups on intention‐to‐treat analysis (*n* = 247) and after propensity matching (*n* = 154).

		Baseline population			After propensity matching		
		UPS (*n* = 138)	pTAT (*n* = 109)	*p*‐value	UPS (*n* = 77)	pTAT (*n* = 77)	*p* Value
**Age**		66 (27–96)	65 (25–94)	0.650	68 (32–96)	66 (29–84)	0.334
**Sex**	Male	116 (84.1%)	89 (81.7%)	0.617	69 (89.6%)	66 (85.7%)	0.462
	Female	22 (15.9%)	20 (18.3%)		8 (10.4%)	11 (14.3%)	
**BMI**		22.95 (16–34.8)	22.79 (16.6–37.02)	0.964	23.4 (16–31.2)	23 (16.6–37.02)	0.519
**Cirrhosis**	Absent	106 (76.9%)	60 (55.1%)	**0.001**	52 (67.5%)	53 (68.8%)	0.863
	Present	32 (23.1%)	49 (44.9%)		25 (32.5%)	24 (31.2%)	
**Portal hypertension**	Absent	130 (94.3%)	90 (82.6%)	**0.004**	70 (90.9%)	69 (89.6%)	
	Present	8 (5.7%)	19 (17.4%)		7 (9.1%)	8 (10.4%)	0.786
**TBS**		9.8 (5.1–30.02)	10.04 (5.39–22.02)	0.516	10.04 (5.1–25.02)	10.04 (5.78–22.02)	1.00
**AFP**		58.29 (1.29–410900)	90 (1.18–1064690)	0.650	35.43 (1.39–410900)	77 (1.18–1064690)	0.519
**ECOG**	0/1	130 (94.2%)	97 (88.9%)		69 (89.6%)	71 (92.2%)	
	2	8 (5.7%)	12 (11.1%)	0.136	8 (10.4%)	6 (7.8%)	0.575
**Viral markers**	Hepatitis B/C	96 (69.5%)	48 (44%)	**0.001**	35 (45.4%)	34 (44.1%)	
	Viral negative	42 (30.5%)	61 (56%)		42 (54.6%)	43 (55.9%)	0.871

*Note*: Values in bold represent a signficant *p* value (<0.05).

Abbreviations: AFP, alpha fetoprotein; BMI, body mass index; ECOG, Eastern Cooperative Oncology Group; pTAT, post‐tranarterial treatment; TBS, tumor burden score; UPS, upfront surgery.

Among the 247 patients of the baseline cohort, 138 underwent UPS and 109 received pTACE [Figure 2].

**FIGURE 3:**
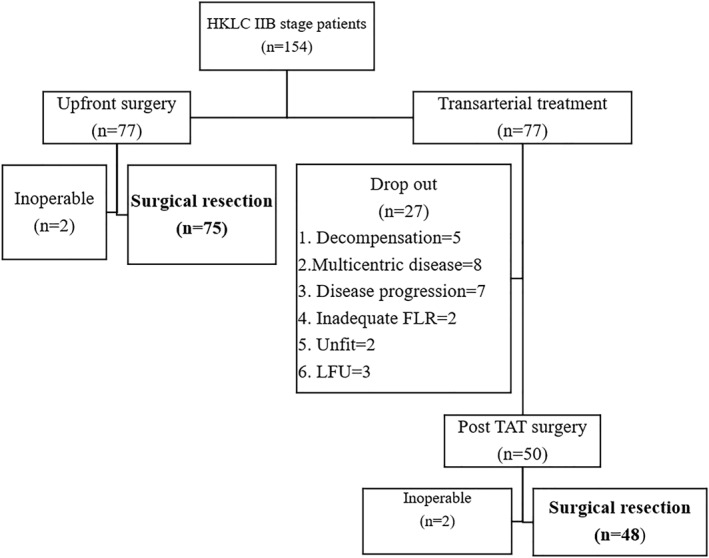
Consort diagram after propensity matching.

After 1:1 propensity matching, there were 77 patients in each group as shown in Figure 3. Of the 77 patients in the UPS group, 75 underwent successful curative resection and two were declared inoperable. Among the 77 patients in the pTACE group, the dropout rate was 35% (27/77), with multicentric disease (*n* = 8 and 29.6%), being the most common reason and 48 patients ultimately underwent successful curative resection, since two patients were deemed inoperable due to the presence of bilobar disease on exploration. The median duration between the last TACE session and surgery was 74 days (range 14–244). The median number of TACE cycles given was 1 (range 1–4). Twenty‐two patients received more than one cycle of TACE.

### 3.1 Perioperative outcomes

Surgical outcomes of UPS and pTACE groups are elaborated in the Supplementary file [Table 1 in Supporting Information S1]. The complication rates in terms of posthepatectomy liver failure (PHLF), posthepatectomy bile leak (PHBL), and posthepatectomy hemorrhage (PHH) were not significantly different.

### 3.2 Overall survival (OS)

**FIGURE 4:**
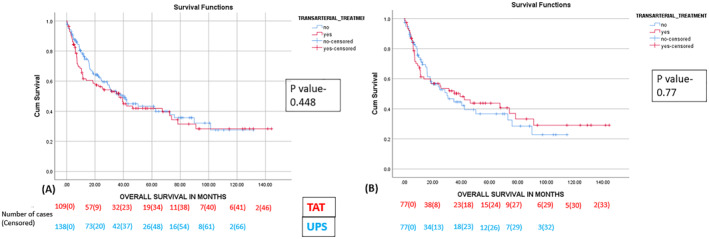
The Kaplan–Meier curve depicting the effect of TACE on OS in the (A) baseline population on intention‐to‐ttreat (*n* = 247) and (B) intention‐to‐treat after propensity matching (*n* = 154).

In the baseline cohort of 247 patients, the median follow‐up was 38.43 months (0.46–144.24). The median OS of the UPS group was 40.4 months (95% CI, 29.57–51.24) as compared to 36.9 months (95% CI, 22.68–51.16) in the pTACE group (*p* value = 0.448) on an intention‐to‐treat analysis [Figure 4A].

In the propensity matched population (*n* = 154), the median follow‐up was 36.4 months (0.46–144.26). The median overall survival of the UPS group and the pTACE group were 30.06 months (95% CI, 13.526–46.597) and 39.26 months (95% CI, 16.74–61.78), respectively (*p* value = 0.77). [Figure 4B].

**FIGURE 5:**
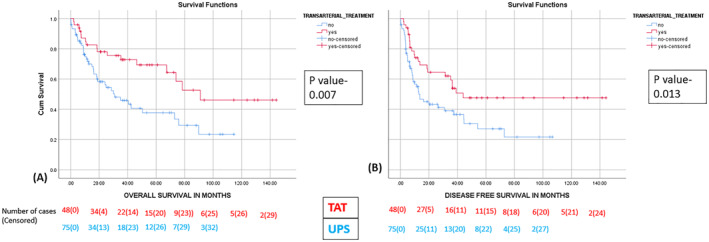
The Kaplan–Meier curve depicting the effect of TACE on (A) OS and (B) DFS in the matched cohort who underwent successful curative resection.

In the same propensity matched population (*n* = 154), on analysis of patients who underwent curative resection, the median overall survival were 30.68 months (95% CI, 14.5–46.8) in the UPS group versus 90.97 months in the pTACE group, respectively (*p* value = 0.006). [Figure 5A].

Multivariate Cox regression analysis of factors affecting OS in the population who underwent successful curative resection, revealed cirrhosis (*p* value = 0.005), lymphovascular invasion (LVI) (*p* value = 0.035), and TACE (*p* value = 0.007) as significant factors affecting OS [Table 2].

**TABLE 2:** Univariate and multivariate analyses of factors affecting OS in the matched cohort who underwent successful curative resection.

		Univariate analysis		Multivariate analysis	
Parameters		Hazard ratio (95% CI)	*p* Value	Hazard ratio (95% CI)	*p* Value
Age		1.00 (0.97–1.02)	0.881		
Sex	Female	1			
	Male	1.54 (0.66–3.66)	0.313		
BMI		0.95 (0.88–1.01)	0.19		
TBS	<7.3	1		1	
	>/ = 7.3	2.63 (1.28–5.42)	**0.008**	2.836 (1.34–5.96)	**0.006**
AFP	<400	1			
	>/ = 400	1.60 (0.93–2.70)	0.08		
ECOG	0/1	1			
	2	1.49 (0.59–3.78)	0.39		
Viral markers	Viral negative	1			
	Hepatitis B/C	1.22 (0.72–2.05)	0.45		
Cirrhosis	Absent	1		1	
	Present	1.80 (1.06–3.07)	**0.029**	2.17 (1.26–3.75)	**0.005**
Portal hypertension	Absent	1			
	Present	1.39 (0.66–2.95)	0.381		
Lymphovascular invasion	Absent	1		1	
	Present	2.18 (1.27–3.76)	**0.005**	1.85 (1.04–3.29)	**0.035**
Perineural invasion	Absent	1			
	Present	2.58 (0.92–7.21)	0.069		
Capsular invasion	Absent	1		1	
	Present	1.57 (1.12–2.21)	**0.009**	1.57 (0.77–3.18)	0.212
Margin	Free	1			
	Involved	5.06 (0.68–37.65)	0.113		
Transarterial treatment	Received	1		1	
	Not received	0.459 (0.25–0.81)	**0.008**	0.44 (0.24–0.79)	**0.007**

*Note*: Values in bold represent a significant *p* value (<0.05).

Abbreviations: AFP, alpha fetoprotein; BMI, body mass index; ECOG, Eastern Cooperative Oncology Group; TBS, tumor burden score.

### 3.3 Disease‐free survival (DFS)

In the baseline cohort of 247 patients, the median DFS of the UPS group was 18.26 months (95% CI, 8.52–28.00) as compared to 13.3 months (95% CI, 5.45–21.15) in the pTACE group (*p* value = 0.663) on an intention‐to‐treat analysis.

In the propensity matched population (*n* = 154), the median DFS of the UPS group and the pTACE group was 13.56 months (95% CI, 7.77–19.36) and 13.76 months (95% CI, 5.38–22.15), respectively (*p* value = 0.77).

Analysis of patients who underwent curative resection showed a median DFS of 13.56 months (95% CI, 4.98–22.15) for the UPS group versus 44.02 months in the pTACE group, respectively (*p* value = 0.013). [Figure 5B].

Multivariate Cox regression analysis of factors affecting DFS in the population who underwent successful curative resection, revealed TBS (*p* value = 0.005), cirrhosis (*p* value = 0.010), capsular invasion (*p* value = 0.018), and TACE (*p* value = 0.022) as significant factors affecting DFS [Table 3]. There was no difference in the recurrence and death patterns among the groups [Supplementary file, Table 2 in Supporting Information S1].

**TABLE 3:** Univariate and multivariate analyses of factors affecting DFS in the matched cohort who underwent successful curative resection.

		Univariate analysis		Multivariate analysis	
Parameters		Hazard ratio (95% CI)	*p* Value	Hazard ratio (95% CI)	*p* Value
Age		0.996 (0.97–1.01)	0.72		
Sex	Female	1			
	Male	1.52 (0.72–3.19)	0.26		
BMI		0.99 (0.93–1.05)	0.908		
TBS	<7.3	1		1	
	>/ = 7.3	2.22 (1.18–4.17)	**0.013**	2.53 (1.31–4.87)	**0.005**
AFP	<400	1			
	>/ = 400	1.59 (0.96–2.63)	0.068		
ECOG	0/1	1			
	2	1.43 (0.62–3.34)	0.397		
Viral markers	Viral negative	1			
	Hepatitis B/C	1.27 (0.79–2.05)	0.315		
Cirrhosis	Absent	1		1	
	Present	2.04 (1.25–3.31)	**0.004**	2.32 (1.41–3.80)	**0.01**
Portal hypertension	Absent	1		1	
	Present	1.96 (1.00–3.86)	**0.04**	1.64 (0.75–3.57)	0.210
Lymphovascular invasion	Absent	1		1	
	Present	1.796 (1.09–2.94)	**0.021**	1.36 (0.80–2.309)	0.249
Perineural invasion	Absent	1			
	Present	2.03 (0.73–5.60)	0.170		
Capsular invasion	Absent	1		1	
	Present	1.67 (1.24–2.26)	**0.001**	2.11 (1.13–3.92)	**0.018**
Margin	Free	1			
	Involved	3.75 (0.51–27.57)	0.194		
Transarterial treatment	Received	1		1	
	Not received	0.529 (0.314–0.87)	**0.013**	0.54 (0.31–0.916)	**0.022**

*Note*: Values in bold represent a significant *p* value (<0.05).

Abbreviations: AFP, alpha fetoprotein; BMI, body mass index; ECOG, Eastern Cooperative Oncology Group; TBS, tumor burden score.

## 4 DISCUSSION

Surgery (liver resection or transplantation) remains the best curative treatment option for HCC.^1^, ^2^ Even successful surgical resections are associated with high rates of intrahepatic recurrences ranging from 50% to 75%.^4^ These intrahepatic recurrences can be early or late.^4^ Early recurrences are the true recurrences of intrahepatic metastases that strongly correlate with tumor characteristics. In contrast, late recurrences tend to be multicentric in origin, which may be related to the condition of the remnant liver. Gao et al. have attributed early recurrences to either preexisting microscopic tumor foci or due to tumor dissemination during surgical manipulation.^4^

Transarterial treatment in the form of TACE has been hypothesized to reduce the early true recurrences due to intrahepatic metastases and prolong survival, whereas others have failed to demonstrate these outcomes.^5^, ^6^, ^17^, ^18^, ^19^ Direct infusion of a lipoidal agent and chemotherapy through the hepatic artery allows a high dose of chemotherapy to be delivered directly to the tumor. Transarterial therapies, such as TACE, have been used for unresectable/locally advanced HCC for tumor downsizing and rendering them amenable to surgical resection. Improved long‐term survival may be achieved in HCC patients who undergo surgical resection after downsizing.^5^, ^6^, ^17^ There is limited evidence of their utility as neoadjuvant treatment in resectable disease.^20^, ^21^ Preoperative TACE can detect micrometastases that are associated with large HCCs.^22^ It also enhances the ability to detect additional small nodules on a CT scan performed 2–3 weeks later, especially in the opposite lobe of cirrhotic livers.^23^

Kairobi et al. concluded that preoperative TACE did not reduce recurrences (local and distant) or improve survival in resectable HCCs.^19^ Zhou et al. conducted a randomized control trial comparing preoperative TACE versus upfront resection and concluded that preoperative TACE was not beneficial in improving survival (DFS and OS) in resectable HCCs.^24^ However, in the present study, majority of patients who received TACE were for downsizing [Figure 2]. Patients who underwent successful curative resection, in the pTACE group, had improved survival that was statistically significant (90.97 vs. 30.68 months with *p* value = 0.006). A pathological complete response was observed in four patients and more than 50% necrosis was seen in 26 patients in post‐TACE resected specimens [Supplementary file, Table 1 in Supporting Information S1]. This marked pathological response seen in 62.5% (30/48) of patients in the pTACE group has likely contributed to the improved survival in resected patients. Another key finding was the lower incidence of LVI in the pTACE group (22.9%) as compared to the UPS group (45.3%), which could be attributed to the effect of treatment. A similar finding was reported by Wang et al., where the microvascular invasion was lower in the TACE + liver resection group.^22^ Preoperative TACE induces massive necrosis that markedly reduces the amount of microvascular invasion in the tumor.^25^, ^26^ Increased incidence of microvascular invasion is often seen in large HCC and is a known poor prognostic factor.^25^, ^26^

One of the major concerns with preoperative TACE is the risk of progression and potential dropouts. Zhou et al. reported a dropout rate of 5% in their group because of liver decompensation or disease progression.^24^ They concluded that these patients had missed the chance of curative resection and cited it as a disadvantage of pTACE. However, it can be argued that patients who suffer liver decompensation post‐TACE have poor functional reserve and are unlikely to tolerate a major hepatectomy thus averting a futile surgery. The dropout rate in the group of patients who received pTACE was 35% in our study with common reasons being liver decompensation, multicentricity, and disease progression [Figure 3]. Also, in the pTACE group, 30% had underlying cirrhosis and up to 10% had features of portal hypertension [Table 1]. Therefore, TACE acted as a preoperative stress test for such patients and thereby helped in the patient selection. Patients who develop progressive disease with a liver‐directed therapy probably have a disease with an inherently aggressive biology and thus would be poor surgical candidates, thereby emphasizing the role of TACE in patient selection.

Some studies have reported that pTACE can make surgery technically difficult because of intraoperative bleeding due to hepatic inflammation, diaphragmatic adhesions, and adhesions with surrounding structures such as the stomach.^27^, ^28^ In the present study, the median duration between TACE and surgery was 74 days (14–244). Nagasue et al. reported that the mean interval between TACE and surgery of 130 days resulted in similar complication rates as in patients who did not receive TACE.^29^ However, in the present study surgical outcomes in terms of PHLF, PHBL, PHH, and Clavien–Dindo scores were not different between the two groups.

This study brings out a fallacy of the HKLC staging system. As per HKLC staging recommendations, all IIB‐stage patients should undergo surgical resection. However, it does not provide clarification on the resectability criteria, for example, large tumors with inadequate FLR and patients with comorbidities requiring optimization before surgery. In the present study, we have included these patients under the subcategory of borderline resectable diseases. These patients need downsizing procedures, such as TACE with or without PVE, to allow augmentation of FLR.

The limitation of this study is its retrospective nature, which is associated with its inherent bias. A propensity matched intention‐to‐treat analysis was performed to reduce that bias. However, though propensity matching was used, unadjusted confounding may still exist as its retrospective data spread over a decade, wherein multiple factors might have influenced treatment decision‐making.

## 5 CONCLUSION

In intermediate‐stage hepatocellular carcinoma (Hong Kong Liver Cancer stage IIB), pTACE can be used to better select patients with borderline resectability. Survival was significantly improved in patients who received pTACE and were able to undergo surgical resection. Thus, it is important to subclassify the intermediate‐stage HCC who would benefit from pTACE and develop strategies to reduce the dropout rates.

## AUTHOR CONTRIBUTIONS

**Kunal Nandy**: Conceptualization; Data curation; Formal analysis; Writing ‐ original draft. **Gurudutt P. Varty**: Data curation; Formal analysis. **Shraddha Patkar**: Conceptualization; Writing ‐ original draft; Writing ‐ review and editing. **Tanvi Shah**: Data curation; Formal analysis. **Kaival Gundavda**: Data curation; Formal analysis; Writing ‐ review and editing. **Kunal Gala**: Writing ‐ review and editing. **Nitin Shetty**: Methodology; Writing ‐ review and editing. **Suyash Kulkarni**: Methodology; Writing ‐ review and editing. **Mahesh Goel**: Conceptualization; Writing ‐ review and editing.

## CONFLICT OF INTEREST STATEMENT

The authors declare that they have no relevant financial or nonfinancial interests to disclose.

## ETHICS STATEMENT

This is an observational study; hence, no ethical approval is required.

## Supporting information

Supporting Information 1

## Data Availability

The datasets generated during and/or analyzed during the current study are available from the corresponding author on reasonable request.
