# Supplementary material for: Role of preoperative transarterial chemoembolization (TACE) in intermediate‐stage hepatocellular carcinoma (Hong Kong liver cancer stage IIB)
Source: World J Surg. 2024 Dec 11;49(2):483–93. doi: 10.1002/wjs.12420 (PMC11798681; doi:10.1002/wjs.12420)
Supplement: Supplementary file 1 — Supporting Information 1 [file WJS-49-483-s001.docx]

**Supplemental Table 1** Surgical Details, outcomes, and pathological outcomes

|  |  | Upfront Surgery  (n=75) | Post TAT  (n=48) | P value |
| --- | --- | --- | --- | --- |
| Resection | Major hepatectomy | 50 | 37 | 0.216 |
|  | Minor hepatectomy | 25 | 11 |  |
| Median surgery duration |  | 240(90-480) | 240(120-495) | 0.480 |
| Median blood loss |  | 1500  (200-11000) ml | 2200  (400-22000) ml | 0.827 |
| Median hospital stay |  | 8(3-34) days | 10(5-44) days | 0.925 |
| PHLF | A | 8 | 9 |  |
|  | B | 26 | 13 | 0.450 |
|  | C | 6 | 2 |  |
| PHBL | A | 5 | 6 |  |
|  | B | 4 | 2 | 0.405 |
|  | C | 0 | 1 |  |
| PHH | B | 1 | 0 |  |
|  | C | 0 | 1 | 0.332 |
| SSI |  | 2 | 1 | 0.838 |
| Clavien Dindo Grade | III | 5 | 4 |  |
|  | IV | 0 | 1 | 0.409 |
|  | V | 6 | 2 |  |
| 90-day mortality |  | 6 | 4 |  |
| Lymphovascular invasion | Absent | 41 | 37 | **0.012** |
|  | Present | 34 | 11 |  |
| Perineural invasion | Absent | 71 | 47 | 0.373 |
|  | Present | 4 | 1 |  |
| Capsular invasion | Absent | 64 | 44 | 0.295 |
|  | Present | 11 | 4 |  |
| Margin | Free | 74 | 48 |  |
|  | Involved | 1 | 0 | 0.422 |
| Pathological response to TAT | PCR | NA | 4 |  |
|  | <50% necrosis | NA | 18 |  |
|  | >50% necrosis | NA | 26 |  |

PHLF-Post hepatectomy liver failure

PHBL-Post hepatectomy bile leak

PHH-Post hepatectomy hemorrhage

SSI-Suture site infection

TAT-transarterial treatment

PCR-Pathological complete response

**Supplemental Table 2** Recurrence and Death Pattern

|  |  | Upfront Surgery  (n=75) | Post TAT(n=48) | P value |
| --- | --- | --- | --- | --- |
| Recurrence pattern | Hepatic | 19 | 9 |  |
|  | Extrahepatic | 5 | 3 | 0.925 |
|  | Both hepatic and extrahepatic | 11 | 7 |  |
| Death | Disease-related | 29 | 13 |  |
|  | Post-surgery | 6 | 2 | 0.172 |
|  | Cirrhosis | 0 | 1 |  |
|  | Others | 5 | 1 |  |

TAT-Transarterial treatment
